# Supplementary material for: Empirical methods for controlling false positives and estimating confidence in ChIP-Seq peaks
Source: BMC Bioinformatics. 2008 Dec 5;9:523. doi: 10.1186/1471-2105-9-523 (PMC2628906; doi:10.1186/1471-2105-9-523)
Supplement: Additional File 1 — A variety of html documents from the USeq web site detailing the available applications, their best usage, output file type descriptions, command line menus, etc. [file 1471-2105-9-523-S1.zip › USeqUserGuides/JavaDoc.zip!/JavaDoc/trans/main/Window.html]

Window


|  |  |  |
| --- | --- | --- |
| **Overview** \| **Package** \| **Class** \| **Use** \| **Tree** \| **Deprecated** \| **Index** \| **Help** | |  |
| **PREV CLASS**   **NEXT CLASS** | **FRAMES**    **NO FRAMES**     **All Classes** |  |
| SUMMARY: NESTED \| FIELD \| CONSTR \| METHOD | DETAIL: FIELD \| CONSTR \| METHOD |  |


---


## trans.main Class Window

```
java.lang.Object
  trans.main.Window
```

**All Implemented Interfaces:**: java.io.Serializable, java.lang.Comparable

---

``` public class Window extends java.lang.Object implements java.lang.Comparable, java.io.Serializable ```

Class to hold information about a window, a defined portion of the genome typically < 1kb.

**See Also:**: Serialized Form

---

**Constructor Summary**

`Window(java.lang.String chromosome,
int start,
int end,
int numberOligos,
double[] scores)`


| **Method Summary** | |
| --- | --- |
| `int` | `compareTo(java.lang.Object obj)` |
| `java.lang.String` | `getChromosome()` |
| `int` | `getNumberOligos()` |
| `double[]` | `getScores()` |
| `double` | `getSortBy()` |
| `int` | `getStart1stOligo()` |
| `int` | `getStartLastOligo()` |
| `boolean` | `overlap(Window other)` |
| `void` | `setScores(double[] scores)` |
| `void` | `setSortBy(double sortBy)` |
| `void` | `setStart1stOligo(int start)` |
| `void` | `setStartLastOligo(int end)` |
| `java.lang.String` | `stringRep(int sizeOfOligo)`             Returns chromosome, start first oligo, start last oligo |

**Methods inherited from class java.lang.Object**

`equals, getClass, hashCode, notify, notifyAll, toString, wait, wait, wait`

**Constructor Detail**

### Window

```
public Window(java.lang.String chromosome,
              int start,
              int end,
              int numberOligos,
              double[] scores)
```


**Method Detail**

### compareTo

```
public int compareTo(java.lang.Object obj)
```

:   **Specified by:**: `compareTo` in interface `java.lang.Comparable`

---


### overlap

```
public boolean overlap(Window other)
```

---


### stringRep

```
public java.lang.String stringRep(int sizeOfOligo)
```

:   Returns chromosome, start first oligo, start last oligo

---


### getChromosome

```
public java.lang.String getChromosome()
```

---


### getStartLastOligo

```
public int getStartLastOligo()
```

---


### getStart1stOligo

```
public int getStart1stOligo()
```

---


### setSortBy

```
public void setSortBy(double sortBy)
```

---


### getSortBy

```
public double getSortBy()
```

---


### setStartLastOligo

```
public void setStartLastOligo(int end)
```

---


### setStart1stOligo

```
public void setStart1stOligo(int start)
```

---


### getScores

```
public double[] getScores()
```

---


### setScores

```
public void setScores(double[] scores)
```

---


### getNumberOligos

```
public int getNumberOligos()
```

:   **Returns:**: Returns the numberOligo positions covered by a window, not all the treatments and controls.


---


|  |  |  |
| --- | --- | --- |
| **Overview** \| **Package** \| **Class** \| **Use** \| **Tree** \| **Deprecated** \| **Index** \| **Help** | |  |
| **PREV CLASS**   **NEXT CLASS** | **FRAMES**    **NO FRAMES**     **All Classes** |  |
| SUMMARY: NESTED \| FIELD \| CONSTR \| METHOD | DETAIL: FIELD \| CONSTR \| METHOD |  |


---
